# Genome-Wide Identification of Resveratrol Intrinsic Resistance Determinants in *Staphylococcus aureus*

**DOI:** 10.3390/antibiotics10010082

**Published:** 2021-01-16

**Authors:** Liping Liu, Hanne Ingmer, Martin Vestergaard

**Affiliations:** Department of Veterinary and Animal Sciences, University of Copenhagen, Stigbøjlen 4, DK-1870 Frederiksberg C, Denmark; lipingliu0430@gmail.com (L.L.); mave@sund.ku.dk (M.V.)

**Keywords:** *Staphylococcus aureus*, resveratrol, intrinsic resistance, DNA damage, SOS response

## Abstract

Resveratrol has been extensively studied due to its potential health benefits in multiple diseases, for example, cancer, obesity and cardiovascular diseases. Besides these properties, resveratrol displays inhibitory activity against a wide range of bacterial species; however, the cellular effects of resveratrol in bacteria remain incompletely understood, especially in the human pathogen, *Staphylococcus aureus*. In this study, we aimed to identify intrinsic resistance genes that aid *S. aureus* in tolerating the activity of resveratrol. We screened the Nebraska Transposon Mutant Library, consisting of 1920 mutants with inactivation of non-essential genes in *S. aureus* JE2, for increased susceptibly to resveratrol. On agar plates containing 0.5× the minimum inhibitory concentration (MIC), 17 transposon mutants failed to grow. Of these, four mutants showed a two-fold reduction in MIC, being the *clpP* protease mutant and three mutants with deficiencies in the electron transport chain (*menD*, *hemB*, *aroC*). The remaining 13 mutants did not show a reduction in MIC, but were confirmed by spot-assays to have increased susceptibility to resveratrol. Several genes were associated with DNA damage repair (*recJ*, *xerC* and *xseA*). Treatment of *S. aureus* JE2 with sub-inhibitory concentrations of resveratrol did not affect the expression of *recJ*, *xerC* and *xseA*, but increased expression of the SOS–stress response genes *lexA* and *recA*, suggesting that resveratrol interferes with DNA integrity in *S. aureus*. Expression of error-prone DNA polymerases are part of the SOS–stress response and we could show that sub-inhibitory concentrations of resveratrol increased overall mutation frequency as measured by formation of rifampicin resistant mutants. Our data show that DNA repair systems are important determinants aiding *S. aureus* to overcome the inhibitory activity of resveratrol. Activation of the SOS response by resveratrol could potentially facilitate the development of resistance towards conventional antibiotics in *S. aureus*.

## 1. Introduction

Resveratrol (3,5,4′-trihydroxystilbene) is a naturally occurring polyphenolic compound, shown to have antioxidant properties. Resveratrol has been widely studied for multiple health-beneficial effects, such as anti-inflammation, anti-carcinogenesis, anti-obesity, anti-aging and cardiovascular protection [1]. Since resveratrol is well-tolerated by humans and, due to the potential health benefits, it is commonly consumed as a dietary supplement [2]. Besides these potential health beneficial effects, resveratrol has inhibitory activity against a range of bacterial species, including methicillin-resistant *Staphylococcus aureus* (MRSA) [3].

Even though several studies have investigated the mechanism of action of resveratrol against different bacterial species, the cellular effects of resveratrol remain incompletely understood and especially underexplored in *S. aureus* [3]. Resveratrol has been shown to bind to the bovine ATP synthase [4], and it partially inhibits both ATP hydrolysis and ATP synthesis in *E. coli* [5]. Inhibition of the ATP synthase by resveratrol has been hypothesized to be the mechanism for potentiation of aminoglycosides, polymyxins and human antimicrobial peptides against *S. aureus* [6,7].

Studies have also shown that resveratrol causes DNA fragmentation in *Escherichia coli* [8,9], leading to increased expression of genes from the SOS–stress regulon [8], that encodes factors involved in cell cycle arrest and DNA repair following DNA damage [10]. Furthermore, *E. coli* mutants lacking genes involved in DNA repair are more sensitive towards resveratrol [9]. The SOS regulon genes are controlled by the LexA repressor that following DNA damage, by for example mitomycin C, undergoes autocleavage in response to binding of RecA to single-stranded DNA [10]. The SOS regulon controls expression of genes involved in DNA repair, recombination and error-prone polymerases [10,11] and activation of the SOS response can promote the selection of antibiotic resistance [12,13,14]. 

Since little is known about the cellular effects of resveratrol on the opportunistic pathogen *S. aureus*, we screened the entire Nebraska Transposon Mutant Library (NTML) for genetic elements that aid *S. aureus* to overcome the inhibitory activity of resveratrol. We identified mutants with defects in DNA repair systems, energy metabolism and protease activity to be more susceptible towards resveratrol.

## 2. Materials and Methods

### 2.1. Strains, Growth Conditions and Medium

We used the *S. aureus* strain JE2 and its derivative mutants from the Nebraska Transposon Mutant Library (NTML), consisting of 1920 single-gene transposon mutants with inactivated non-essential genes [15]. All strains were grown in tryptic soy broth (TSB, Oxoid, Hampshire, UK) or on tryptic soy agar (TSA, Oxoid) at 37 °C. Chemicals used in this study include, resveratrol (Santa Cruz Biotechnology, Santa Cruz, CA, USA), erythromycin (Sigma, St. Louis, MO, USA), rifampicin (Sigma) and mitomycin C (Sigma).

### 2.2. Minimum Inhibitory Concentration

The minimum inhibitory concentrations (MIC) for resveratrol and mitomycin C were determined using the broth microdilution assay in 96-well plates. Overnight cultures of *S. aureus* were diluted in physiological saline (0.9% NaCl) to reach a turbidity of 0.5 McFarland (Sensititre^®^ Nephelometer and the Sensititre^®^ McFarland Standard). Bacterial suspensions were adjusted to 5 × 10^5^ colony forming units (CFU)/mL in TSB broth containing twofold dilutions of resveratrol or mitomycin C in a final volume of 100 µL. The plates were incubated for 24 h at 37 °C without shaking. MIC was defined as the concentration of the agents that completely prevented visible growth. All experiments were performed in duplicate.

### 2.3. Screening NTML for Increased Susceptibility towards Resveratrol

Screening out mutants with increased susceptibility was performed as previously described [16]. In brief, material from the frozen NTML stock was transferred directly with a Deutz 96 cryoreplicator from the 96-well microtiter plates onto TSA plates supplemented with 128 µg/mL resveratrol (0.5× MIC) and 5 µg/mL erythromycin (the transposon used to create the NTML contains the erythromycin-resistance gene, *ermB* [15]). The plates were incubated at 37 °C for 24 h and visually inspected for lack of growth of individual mutants.

### 2.4. Confirmation Susceptibility of Mutants towards Resveratrol

Overnight cultures of *S. aureus* JE2 and mutants displaying inability to grow on plates supplemented with resveratrol at 0.5× MIC were diluted 1:1000 and grown to exponential phase (OD_600_ = 0.2). The growing culture were diluted 10^−1^-, 10^−2^-, 10^−3^- and 10^−4^-fold, and then 10 µL of each dilution were spotted on TSA plates supplemented with 0.5× MIC resveratrol and on TSA plates without resveratrol as a growth control. The *S. aureus* JE2 strain and two random mutants were added as control strains. All plates were incubated at 37 °C for 24 h and then inspected for growth at the spots. We scored resveratrol susceptibility according to:

+: inhibited growth in 10^−4^ dilution;

++: inhibited growth in 10^−3^ and 10^−4^ dilution;

+++: inhibited growth in 10^−2^, 10^−3^ and 10^−4^ dilution;

++++: inhibited growth in 10^−1^, 10^−2^, 10^−3^, 10^−4^ dilution.

### 2.5. Gene Expression by Real Time Quantitative Polymerase Chain Reaction (RT-qPCR)

Overnight cultures of *S. aureus* JE2 were diluted 1000-fold in fresh TSB medium and grown to OD_600_ = 0.2 with shaking at 37 °C. Then, 5 mL aliquots were transferred to 100 mL Erlenmeyer flasks and treated with resveratrol (64 or 128 μg/mL) or mitomycin C (0.2 μg/mL). Untreated cultures were used as the reference condition. Treated and untreated cells were incubated with shaking at 37 °C and collected after 1 h. The RNeasy Mini Kit (Qiagen, Hilden, Germany) was used to extract total RNA according to the guidelines of the manufacturer. RNA samples were purified by DNA-free Kit (Invitrogen, Carlsbad, CA, USA) to remove contaminating genomic DNA. Then, cDNA was prepared using High Capacity cDNA Reverse Transcription Kit (Thermo Fisher Scientific, Waltham, MA, USA). FastStart Essential DNA Green Master (Roche, Basel, Switzerland) was used to perform RT-qPCR on a LightCycler 96 (Roche). Expression levels of *lexA*, *recA*, *recJ*, *xerC* and *xseA* were measured and *gmk* was used as the reference gene to normalize gene expression (primer sequences can be viewed in Table 1). At least three biological replicates and two technical replicates were performed for each sample. The 2-*^ΔΔCT^* method was used to calculate the normalized fold change between untreated and treated cells [17].

### 2.6. Quantification of Rifampicin Resistant Mutants

Overnight cultures of *S. aureus* JE2 were diluted 1000-fold in 2 mL fresh TSB in 14 mL Falcon tubes supplemented with resveratrol (either 64 μg/mL or 128 μg/mL) or without (TSB only). The cultures were grown for 24 h at 37 °C with shaking (180 rpm). Total colony forming units (CFU) were determined on TSA plates and rifampicin resistant mutant CFU were determined on TSA supplemented with rifampicin (5 μg/mL). Plates were incubated at 37 °C and total CFU and rifampicin resistant mutants were counted following 24 h growth. The mutation frequency of rifampicin resistance was determined as the number of rifampicin resistant mutants divided by the total CFU count. The frequencies reported are the mean of 9 independent experiments.

### 2.7. Statistics

Data were analysed using GraphPad Prism 8 (GraphPad Software Inc., San Diego, CA, USA). Data concerning rifampicin resistance frequency were analysed using one-way analysis of variance with log-transformed datasets and with a post-hoc analysis of Dunnett’s multiple comparison tests. 

Data for the qPCR were analysed using one-sample *t*-tests. The null hypothesis (H_0_) assumes that the difference between the true mean (μ) and the comparison value is equal to zero (H_0_: μ = 1). The two-tailed alternative hypothesis (H_1_) assumes that the difference between the true mean (μ) and the comparison value is not equal to zero (H_1_: μ ≠ 1).

For all statistical analyses, then *p* < 0.05 values were considered significant and the degrees of statistical significance are presented as ★ *p* < 0.05, ★★ *p* < 0.01, and ★★★ *p* < 0.001.

### 2.8. Ethical Approval

Not required.

## 3. Results and Discussion

### 3.1. The Resveratrol Intrinsic Resistome

**Figure 1:**
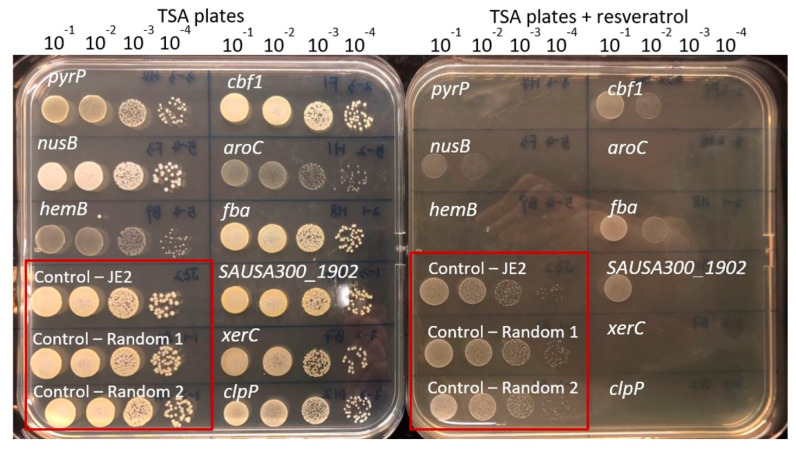
Confirmation of screened out mutants with increased susceptibility towards resveratrol. The growing cells were diluted 10^−1^, 10^−2^, 10^−3^ and 10^−4^ and fold then spotted on the tryptic soy agar (TSA) plates supplemented with resveratrol (128 µg/mL) or on drug-free TSA plates. The plates shown contain the same mutants and are provided as an example of the assay.

To identify intrinsic resistance mechanisms against resveratrol in *S. aureus*, we screened the entire NTML for mutants displaying lack of growth on agar plates supplemented with resveratrol at 128 µg/mL, a concentration that is half of the MIC. A total of 17 mutants were unable to grow on agar plates supplemented with resveratrol at this concentration (Table 2 and Figure 1). The MIC for resveratrol was subsequently measured for the identified mutants, and here four mutants (*clpP*, *hemB*, *aroC* and *menD*) were confirmed to be 2-fold more susceptible than the wild type (Table 2). Next, we assessed resveratrol susceptibility of the identified mutants by spotting serial dilutions of the mutants on agar plates supplemented with resveratrol (0.5× MIC) and several additional genes involved in DNA repair (*recJ*, *xseA* and *xerC*) and the uracil permease (*pyrP*) were among the mutants with the greatest increase in susceptibility using this assay (Figure 1).

### 3.2. Small Colony Variants Are More Susceptible towards Resveratrol

Three of the mutants we screened out being more susceptible to resveratrol are associated with the electron transport chain (*aroC*, *hemB* and *menD*). The *menD* gene is part of the biosynthesis pathway for menaquione [18], while the *aroC* gene encodes for the chorismate synthase that is the final enzyme in the shikimate pathway needed for the synthesis of chorismate, a precursor molecule for the biosynthesis pathway of menaquinone [19]. The *hemB* gene is part of the biosynthesis pathway for the heme group in cytochromes [18].

The three mutants have previously been shown to have reduced membrane potentials and all appear as small colony variants (SCV) on agar plates [20]. Since resveratrol inhibits bovine and *E. coli* ATP synthases [4,5], it can be speculated that the dual interference with the electron transport chain and ATP synthase activity is deleterious for the cells due to energy depletion. In support of this, Langlois and colleagues recently showed that the increased sensitivity of SCVs to the ATP synthase inhibitor tomatidine, was due to a critical drop in membrane potential, which was not observed in WT strains [21]. Collectively, these data indicate that inhibition of the ATP synthase could be a strategy for targeting electron-transport-chain SCVs.

### 3.3. DNA Damage Repair

Three mutants with inactivation of genes related to DNA damage repair, *recJ*, *xseA* and *xerC*, were more susceptible to resveratrol based on the spot assay (Table 2). Both *recJ* and *xseA* encode for exonucleases that degrade single-stranded DNA, respectively RecJ and Exonuclease VII (XseAB, subunits encoded by *xseA* and *xseB*) [22]. The *xerC* gene encodes for a site-specific recombinase XerC, where the homologue in *E. coli* has previously been shown to resolve chromosome dimers for efficient partitioning into daughter cells [23]. No mutant exists in NTML with a transposon in *xseB*.

Our screen corroborates a previous study of *E. coli*, showing that mutants with inactivation of genes involved in repair of DNA damage were more susceptible to resveratrol [9]. In *E. coli*, resveratrol causes DNA damage [8,9] and sub-inhibitory concentrations of the agent activates the SOS–stress response [8]. The mechanism of resveratrol-mediated DNA damage currently remains incompletely understood. Some studies have shown that resveratrol increases reactive oxygen species (ROS) formation, which may be the cause of DNA damage [8,24,25]. Although resveratrol stimulated ROS formation, both *E. coli* studies showed that ROS formation was not associated with the growth inhibitory activity of resveratrol [8,24], which contrasts the importance of resveratrol-stimulated ROS-formation in mediating cell death in *Salmonella typhimurium* [25]. Yet, another study showed that resveratrol decreases ROS levels stimulated by oxolinic acid, a DNA gyrase inhibitor [26]. It, therefore, remains inconclusive whether resveratrol causes DNA damage by inducing oxidative stress in bacterial cells or if the effect is directly on the DNA. 

Previously, *xseA* and *xerC* have also been found as ciprofloxacin intrinsic resistance genes in *S. aureus* JE2 [27]. Ciprofloxacin is an inhibitor of the bacterial DNA gyrase and topoisomerase IV, which leads to a halt in replication and eventually in the breakage of the DNA strands [28]. Inactivation of *xseA* also sensitizes *E. coli* to ciprofloxacin, while the *recJ* mutant in *E. coli* is more susceptible to the DNA damaging agents, nitrofurantoin and metronidazole [29]. Contrarily, *xerC* is not part of the intrinsic resistome towards neither ciprofloxacin, nitrofurantoin nor metronidazole in *E. coli* [29]. 

Since *xseA*-, *recJ*- and *xerC*-mutants are more susceptible to various DNA damaging agents, it indicates that resveratrol by an unknown mechanism could also interfere with DNA integrity in *S. aureus*.

We also identified the *clpP* gene, encoding the proteolytic subunit of the ClpXP two-component protease [30], to be more susceptible towards resveratrol (Table 2). Resveratrol might cause protein damage by stimulating ROS formation, which is toxic to all macromolecules [31] and ClpP proteolytic complexes are important in the degradation of denatured proteins [30]. Furthermore, a *clpP* mutant is more susceptible towards oxidative stress conferred by hydrogen peroxide [30]. A possible link between ClpP and resveratrol susceptibility could be that ClpP interferes with activation of the SOS–stress response [32]. 

### 3.4. Resveratrol Activates the SOS–Stress Response 

Since it has been reported that resveratrol activates the SOS–stress response in *E. coli* [8], we used qPCR to measure the expression of the screened out genes, *recJ*, *xerC* and *xseA*, as well as *lexA* and *recA* that control activation of the SOS–stress response [10,11]. 

**Figure 2:**
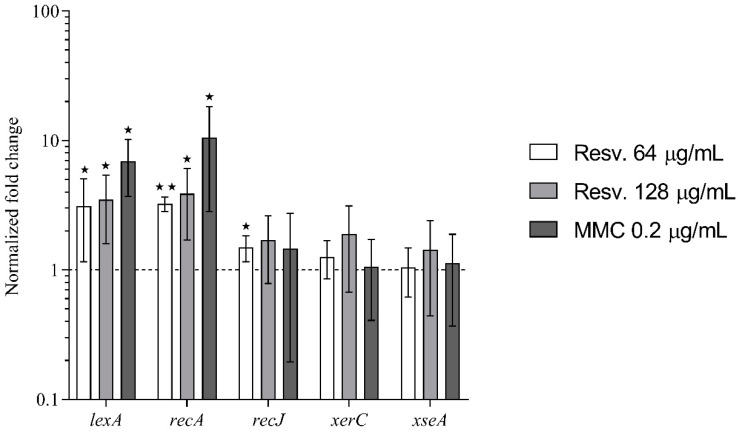
Effect of resveratrol on the expression of DNA repair– and SOS–stress response genes. qPCR was used to measure alterations in gene expression of *lexA*, *recA*, *recJ*, *xerC* and *xseA* in *S. aureus* JE2 cells grown in the presence of resveratrol (0.25× or 0.5× MIC) or mitomycin C (0.5× MIC) relative to untreated cultures. Each column represents the mean of at least three biological replicates. Error bars represent 95% confidence intervals. ★ *p* < 0.05 and ★★ *p* < 0.01.

Resveratrol at sub-inhibitory concentrations (0.25× MIC and 0.5× MIC) increased the expression of *lexA* and *recA* (Figure 2), but not to the same extent as observed for mitomycin C-treated cells (0.5× MIC). Resveratrol and mitomycin C contrarily had no effect on the expression of *recJ, xerC* and *xseA* (<2-fold changes in expression).

These data show that resveratrol activates the SOS–stress response in *S. aureus*, similarly to what previously has been shown in *E. coli* [8]. 

### 3.5. Resveratrol Induces Rifampicin Resistant Mutations

Besides controlling the expression of DNA repair and recombination proteins, the SOS regulon also regulates the expression of error-prone DNA polymerases [10,11]. In *S. aureus* the error-prone polymerase V, encoded by the gene *umuC*, is highly up-regulated upon SOS response activation [11]. Reduced replication fidelity in our study was monitored by selecting for rifampicin resistance, which generally arises by spontaneous mutations in *rpoB* [33].

**Figure 3:**
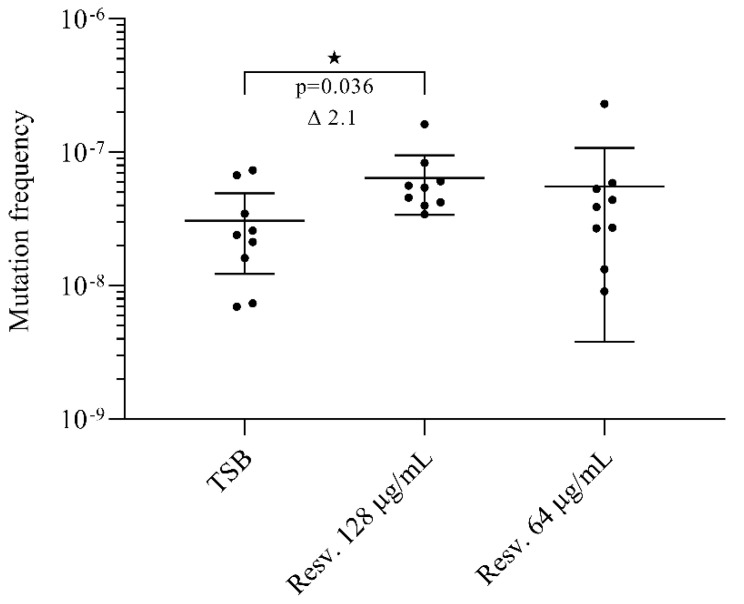
Effect of resveratrol on the recovery of rifampicin resistant mutants. Cells were grown with resveratrol (0.25× or 0.5× MIC) or without for 24 h. Rifampicin resistant mutants were selected on agar plates supplemented with rifampicin (5 µg/mL) and total colony forming units (CFU) were determined on drug-free agar plates. The mutation frequency was calculated as CFU_rif_/CFU_drug free_. For each condition, we assessed the mutation frequency for nine biological replicates. The results are shown as individual measurements and with the mean presented. Error bars represent 95% confidence intervals. ★ *p* < 0.05.

The frequency of rifampicin resistant mutants in un-exposed cultures was 3 × 10^−8^ (Figure 3). Exposure to resveratrol at 0.5× MIC increased the frequency of rifampicin resistant mutants by 2.1-fold (*p* = 0.036), whereas resveratrol at 0.25× MIC did not significantly affect recovery of rifampicin resistant mutants. 

Short-term resveratrol-exposure (0.5× MIC) has been shown to increase the recovery of rifampicin-resistant mutants in *E. coli* by 2-fold, but this effect was contrarily not observed in *S. aureus* in this study [26]. Interestingly, resveratrol in combination with other antibiotics, especially the DNA damaging agent ciprofloxacin, amplified the formation of rifampicin-resistant mutants [26]. 

These data indicate that resveratrol potentially can promote the selection of antibiotic resistant mutants.

## 4. Conclusions

In this work, we provide the first whole genome screen to identify genes that aid *S. aureus* in coping with the stresses conferred by resveratrol. Four mutants (*clpP*, *hemB*, *aroC* and *menD*) displayed a 2-fold reduction in MIC. In contrast, genetic inactivation or chemical inhibition of drug efflux pumps caused a greater reduction in resveratrol MIC in different Gram-negative bacteria, including *E. coli*, *Pseudomonas aeruginosa* and *Arcobacter* species [34,35,36], than we observed for any of the mutations in *S. aureus*. These differences could suggest that the outer membrane and active efflux in Gram-negative bacteria prevents resveratrol from reaching its site of action. Even though the mechanism of action of resveratrol remain incompletely understood, our study supports previous work in *E. coli* [8], showing that resveratrol activates the expression of the SOS–stress response in both Gram-positive and Gram-negative species. We also show that resveratrol promoted the formation of rifampicin resistant mutants, suggesting that concurrent exposure to resveratrol and certain antibiotics could potentially facilitate the emergence of antibiotic resistance to conventional antibiotics.

## Figures and Tables

**Table 1:** Primer sequences for real-time qPCR.

Gene	Forward Primer	Reverse Primer
*lexA*	GTTCCTATTACCGCAGTA	TACCAGCCTCAATCATAC
*recA*	GAGAAATCTTTCGGTAAAGGT	GTGAAGCGCTACTGTTGTCTTACC
*recJ*	ACTATCACAGAAGAAGCAATGG	CGAAGCAACAATACCTAAGACA
*xerC*	TTGGTGCTTATTGTAGAC	GGATGAATCTCACTTACG
*xseA*	GTGTAGATACCATTATTGTAGG	ATGACCAACTGCTGATAT
*gmk*	CCATCTGGAGTAGGTAAAGG	CTACGCCATCAACTTCAC

**Table 2:** Screened out genes with increased susceptibility towards resveratrol.

Gene Entry	Gene Name	Gene Product Description	MIC (µg/mL)	Inhibitory Level
*S. aureus* JE2 (WT)			256	
SAUSA300_1592	*recJ*	Single-stranded-DNA-specific exonuclease RecJ	256	+++
SAUSA300_1472	*xseA*	Exodeoxyribonuclease VII, large subunit	256	++++
SAUSA300_0752	*clpP*	ATP-dependent Clp protease proteolytic subunit	128	++++
SAUSA300_1092	*pyrP*	Uracil permease	256	++++
SAUSA300_1357	*aroC*	Chorismate synthase	128	++++
SAUSA300_1615	*hemB*	Delta-aminolevulinic acid dehydratase	128	++++
SAUSA300_0946	*menD*	2-succinyl-6-hydroxy-2,4-cyclohexadiene-1-carboxylate synthase	128	++++
SAUSA300_1145	*xerC*	Tyrosine recombinase xerC	256	++++
SAUSA300_1573		Holliday junction resolvase-like protein	256	++
SAUSA300_1473	*nusB*	Transcription antitermination protein NusB	256	++
SAUSA300_2079	*fba*	Fructose bisphosphate aldolase	256	++
SAUSA300_1791	*cbf1*	Cmp-binding-factor 1	256	+
SAUSA300_0947		Hydrolase, alpha/beta hydrolase fold family	256	+
SAUSA300_1558	*mtnN*	5′-methylthioadenosine/S-adenosylhomocysteine nucleosidase	256	+
SAUSA300_1359		Polyprenyl synthetase	256	+
SAUSA300_1902		Conserved hypothetical protein	256	+++
SAUSA300_1322		Hypothetical protein	256	++

Exponentially growing cells were diluted 10^−1^-, 10^−2^-, 10^−3^- and 10^−4^-fold and then spotted on TSA plates supplemented with resveratrol (128 µg/mL). +: inhibited growth in 10^−4^ dilution. ++: inhibited growth in 10^−3^ and 10^−4^ dilution. +++: inhibited growth in 10^−2^, 10^−3^ and 10^−4^ dilution. ++++: inhibited growth in 10^−1^, 10^−2^, 10^−3^, 10^−4^ dilution.

## Data Availability

The data presented in this study are available on request from the corresponding author.
